# Partial characterization and evaluation of antioxidant and antibacterial activities of a water soluble polysaccharide isolated from *Euphorbia hebecarpa* roots

**DOI:** 10.1038/s41598-026-42880-7

**Published:** 2026-04-20

**Authors:** Elham Ahmadi Juybari, Mahdi Moridi Farimani, Mojtaba Asadollahi

**Affiliations:** 1 Department of Chemistry, Faculty of Sciences, Golestan University, Gorgan, Iran; 2 Department of Phytochemistry, Medicinal Plants and Drugs Research Institute, Shahid Beheshti University, Evin, Tehran Iran; 3 Department of Natural Sciences, Mid Sweden University, Sundsvall, Sweden

**Keywords:** Biological activity, Carbohydrate, Euphorbiaceae, Identification, Purification, Biochemistry, Biological techniques, Biotechnology, Chemistry, Drug discovery, Microbiology

## Abstract

*Euphorbia hebecarpa* has been valued in traditional Iranian medicine; however, its carbohydrate composition has remained largely unexplored. The present study aimed to isolate, identify, and investigate selected biological properties of a carbohydrate fraction (EHC-1) from *E. hebecarpa*. EHC-1 was extracted using hot water extraction, followed by a two-step chromatographic procedure utilizing DEAE-52 cellulose and Sephadex G-200 columns. Partial structural characterization was performed using gas chromatography-mass spectrometry (GC-MS), high-performance liquid chromatography- refractive index (HPLC-RID), Fourier transform infrared spectroscopy (FT-IR), and gas chromatography-flame ionization detection (GC-FID). In addition, X-ray diffraction (XRD) confirmed the semi-crystalline nature of the polysaccharide, and thermogravimetric analysis (TGA) was employed to evaluate its thermal stability. Compositional analyses identified EHC-1 as a polysaccharide with an average molecular weight of approximately 148 kDa. The relative monosaccharide composition comprised glucose (51.50%), fructose (35.70%), galactose (5.79%), arabinose (5.86%), and galacturonic acid (1.13%), as determined by the corrected peak area method incorporating relative response factors (RRFs). In vitro assays revealed that EHC-1 demonstrated concentration-dependent antioxidant activity in DPPH, ABTS, and hydroxyl radical scavenging assays, with relatively higher efficacy against hydroxyl radicals (IC_50_ = 2.43 ± 0.066 mg/mL). Furthermore, EHC-1 showed moderate antibacterial effects against both *Escherichia coli* and *Staphylococcus aureus* strains, with stronger effects against the latter.

## Introduction

Carbohydrates, particularly polysaccharides, are among the most crucial classes of biological macromolecules, playing vital roles in both metabolic and structural processes in living organisms. These macromolecules, composed of monosaccharide units, exhibit a wide range of functions in nature^1^. In plants, carbohydrates not only serve as the primary energy source but also play key roles in cell wall structure and nutrient storage^2^. In addition to these fundamental roles, bioactive carbohydrates have been reported to exhibit various biological activities, including antitumor, antioxidant, immunomodulatory, prebiotic, and antibiotic effects^3–6^.

The genus *Euphorbia*, a member of the Euphorbiaceae family, is one of the most extensive and diverse plant genera, encompassing over 2,000 recognized species. These species are widely distributed across different continents including Africa, America (Central and South), and Southwest Asia^7,8^. In Iran, approximately 90–92 taxa of *Euphorbia* have been reported, of which 19–21 are considered endemic^9^. The phytochemical diversity of this genus is remarkable, as Euphorbia species are known to produce a wide range of metabolites, including terpenoids (triterpenoids, diterpenes), flavonoids, polyphenols, alkaloids, carotenoids, fatty acids, proteins and carbohydrates^10–12^. Owing to these bioactive constituents, Euphorbia species have been extensively used in both traditional and modern medicine for treating a broad spectrum of ailments, such as gastrointestinal and respiratory disorders, dermatological issues, migraines, inflammatory diseases, intestinal parasitic infections, warts, gonorrhea, snakebites, leprosy, kidney stones, and cancer^10,13^.

The pharmacological potential of specific *Euphorbia* species has been confirmed in several studies. For instance, *n*-hexane extracts of *E. hebecarpa* and *E. petiolata* showed significant antitumor activity by inducing apoptosis in cancer cells and stimulating cytokine secretion from T-helper subsets, thereby improving immune responses against cancer cells. These findings suggest their potential as natural immunomodulatory agents for cancer therapy^14^. In addition, ethanolic extracts of *E. hebecarpa* demonstrated notable antibacterial properties, effectively inhibiting the growth of pathogenic planktonic bacteria and preventing biofilm formation. Notably, these extracts reduced metabolic activity within established biofilms, particularly those formed by *Staphylococcus aureus* and *Bacillus cereus*^15^. Additional phytochemical insights into *E. hebecarpa* have been obtained through essential oil analysis. Hydrodistillation of *E. hebecarpa* collected from Kerman, Iran, yielded 0.2% (w/w) essential oil, in which 28 compounds were identified, accounting for 97.6% of the total oil composition. The major components included α-bisabolol, cis-cadin-4-en-7-ol, trans-piperitol, cis-p-menth-2-en-1-ol, and trans-p-menth-2-en-1-ol, with oxygenated sesquiterpenes representing 59.3% of the identified compounds^16^.

Alongside these phytochemicals, polysaccharides from *Euphorbia* species have attracted increasing scientific interest. Numerous studies have reported the extraction and purification of polysaccharides from different species of this genus. For instance, Liu et al. isolated a polysaccharide from the roots of *E. fischeriana* composed of mannose, glucose, arabinose, galactose, xylose, and rhamnose in molar ratios of 1:21:8:3:1:5, respectively^17^. Similarly, Bao et al. reported the purification of a polysaccharide from *E. himalayensis* roots, which mainly consisted of glucose, galactose, and mannose^18^. In another study, Xiang et al. extracted a polysaccharide from *E. humifusa* that was primarily composed of galactose, glucose, and glucuronic acid^19^.

Several carbohydrates isolated from *Euphorbia* species have demonstrated antioxidant activities. For example, a polysaccharide isolated from *E. himalayensis* roots showed significant free radical scavenging capacity against 1,1-diphenyl-2-picrylhydrazyl (DPPH) (224 µg/mL) and superoxide radicals (320 µg/mL). This carbohydrate was also reported to enhance the activities of superoxide dismutase (SOD) and reduced glutathione (GSH), while decreasing malondialdehyde (MDA) levels in human umbilical vein endothelial cells (HUVECs), thereby confirming its antioxidant efficacy^18^. Similarly, polysaccharides extracted from *E. bivonae* leaves showed strong radical scavenging activity, with DPPH and 2,2'-azino-bis (3-ethylbenzothiazoline-6-sulfonic acid) (ABTS) inhibition rates of 81.56% and 84.32%, respectively, at 1 mg/mL. These polysaccharides also enhanced the activities of SOD, catalase (CAT), glutathione peroxidase (GSH-Px), and GSH in H_2_O_2_-exposed HEK293 cells^20^.

Despite the recognized importance of *E. hebecarpa* in traditional Iranian medicine, its carbohydrate profile remains underexplored. This gap in knowledge results in limited understanding of pharmacological mechanisms and therapeutic potential. The present study aims to address this gap by isolating and structurally characterizing the primary water-soluble polysaccharide from *E. hebecarpa*. The isolation methodology involved sequential chromatographic purification using DEAE ion-exchange and Sephadex columns, followed by structural elucidation through a combination of analytical techniques, including gas chromatography with flame ionization detection (GC-FID), gas chromatography–mass spectrometry (GC–MS), high-performance gel-permeation chromatography (HPGPC), and Fourier-transform infrared (FTIR) analyses. Finally, the biological efficacy of the isolated polysaccharide was assessed by measuring its antioxidant capacity against DPPH, hydroxyl (OH), and ABTS radicals, as well as its antibacterial effects against *S. aureus* and *Escherichia coli*.

## Results

### Isolation and purification of EHC-1

**Fig. 1:**
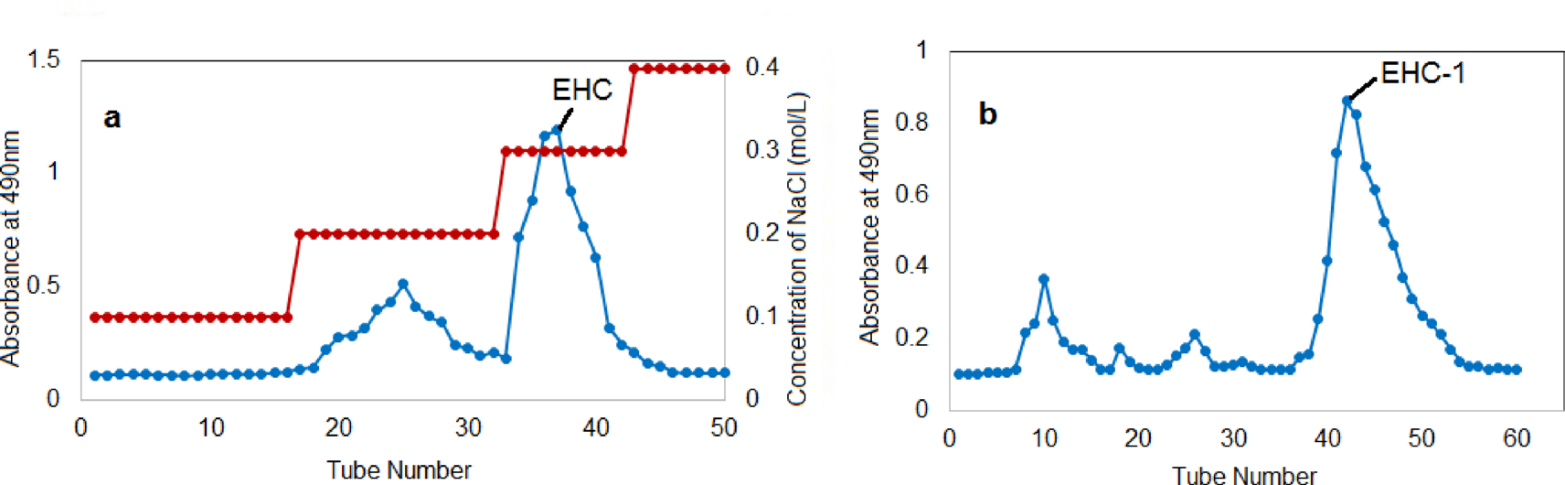
Carbohydrate fractions profile from: (**a**) DEAE-A52 cellulose column; (**b**) Sephadex G-200 column.

Crude *E. hebecarpa* carbohydrate (CEHC) was successfully obtained from *E. hebecarpa* roots with a yield of 1.4% (w/w) using hot water extraction (75 °C), followed by ethanol precipitation (96%) and deproteinization via the Sevag technique^21^. CEHC was further purified using a sequential chromatographic strategy employing DEAE- Cellulose A52 and Sephadex G-200 columns. Fractionation on the DEAE-cellulose A52 column yielded two carbohydrate-containing peaks, which were detected using the phenol–sulfuric acid assay (Fig. 1a). Fractions corresponding to the second peak were subsequently subjected to the Sephadex G-200 column for additional purification. The major fraction obtained from the Sephadex G-200 profile (EHC-1) (Fig. 1b) was collected, pooled, and freeze-dried.

### Analysis of EHC-1: molecular weight determination

**Fig. 2:**
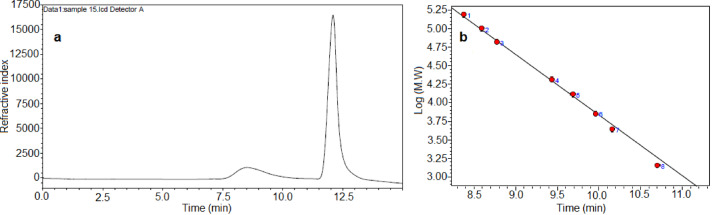
(**a**) HPGPC chromatogram of EHC-1; (**b**) Standard calibration curve.

The total carbohydrate content of EHC-1 was 96.1 ± 0.9%, while its uronic acid content was quantified at 1.47% using the *m*-hydroxybiphenyl colorimetric method. Furthermore, the DNS assay confirmed the absence of reducing sugars. The molecular weight of EHC-1 was determined based on its elution peak in the HPGPC chromatogram (Fig. 2a). Using a standard calibration curve (Fig. 2b), the average molecular weight of EHC-1 was calculated at 148 kDa. The polydispersity index (PDI) was found to be 3.44, indicating a relatively broad molecular weight distribution.

### Analysis of EHC-1: UV–Vis and FT-IR

**Fig. 3:**
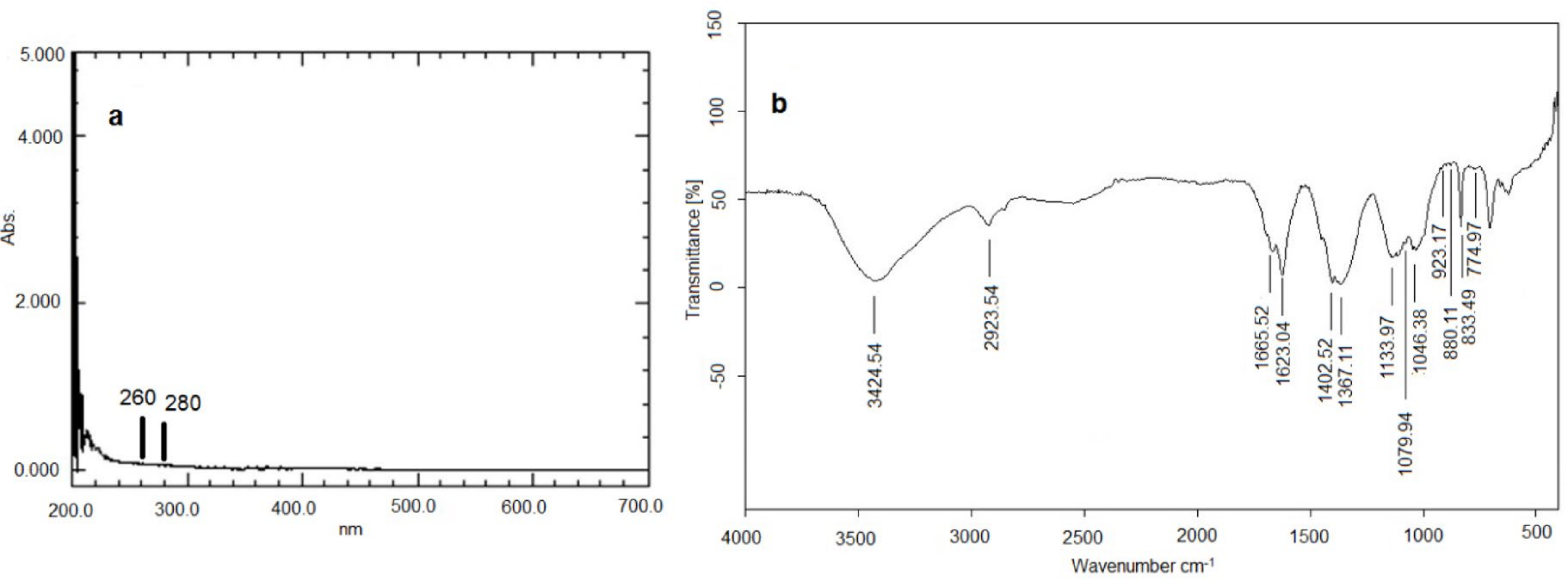
Spectroscopic analysis of EHC-1: (**a**) UV–Vis spectrum; (**b**) FT-IR spectrum.

The UV–Vis spectrum of EHC-1 (Fig. 3a) showed no detectable absorption peaks at 260 nm or 280 nm. The absence of these characteristic peaks confirms that EHC-1 is free of nucleic acids and proteins, validating the high purity of the isolated polysaccharide^22^. Analysis of the FT-IR spectrum (Fig. 3b) revealed a broad absorption band at 3424.54 cm^−1^ attributed to hydroxyl stretching vibrations. A characteristic C–H stretching vibration frequency was observed at 2923.54 cm^−123^. Absorption peaks around 1600 cm^−1^ suggested the presence of carboxylate (COO^−^) groups^24^. The bands at 1367.11 cm^−1^ and 1402.52 cm^−1^ were assigned to C–H deformation vibrations^25^. Additionally, the signals at 1046.38 cm^−1^ and 1079.94 cm^−1^ were assigned to the C–O stretching vibrations of alcoholic groups. An absorption peak at 1133.97 cm^−1^ could indicate the potential presence of ether linkages^18^. The bands at 880.11 cm^−1^ and 833.49 cm^−1^ were tentatively associated with β- and α-anomeric configurations, respectively. Furthermore, the absorption peaks at 774.97 cm^−1^ and 923.17 cm^−1^ suggested the possible presence of pyranose and furanose rings^25,26^. However, these FT-IR-based assignments regarding ring conformations and glycosidic linkages are preliminary and remain tentative in the absence of advanced NMR spectroscopic data.

### Analysis of EHC-1: monosaccharide composition

**Fig. 4:**
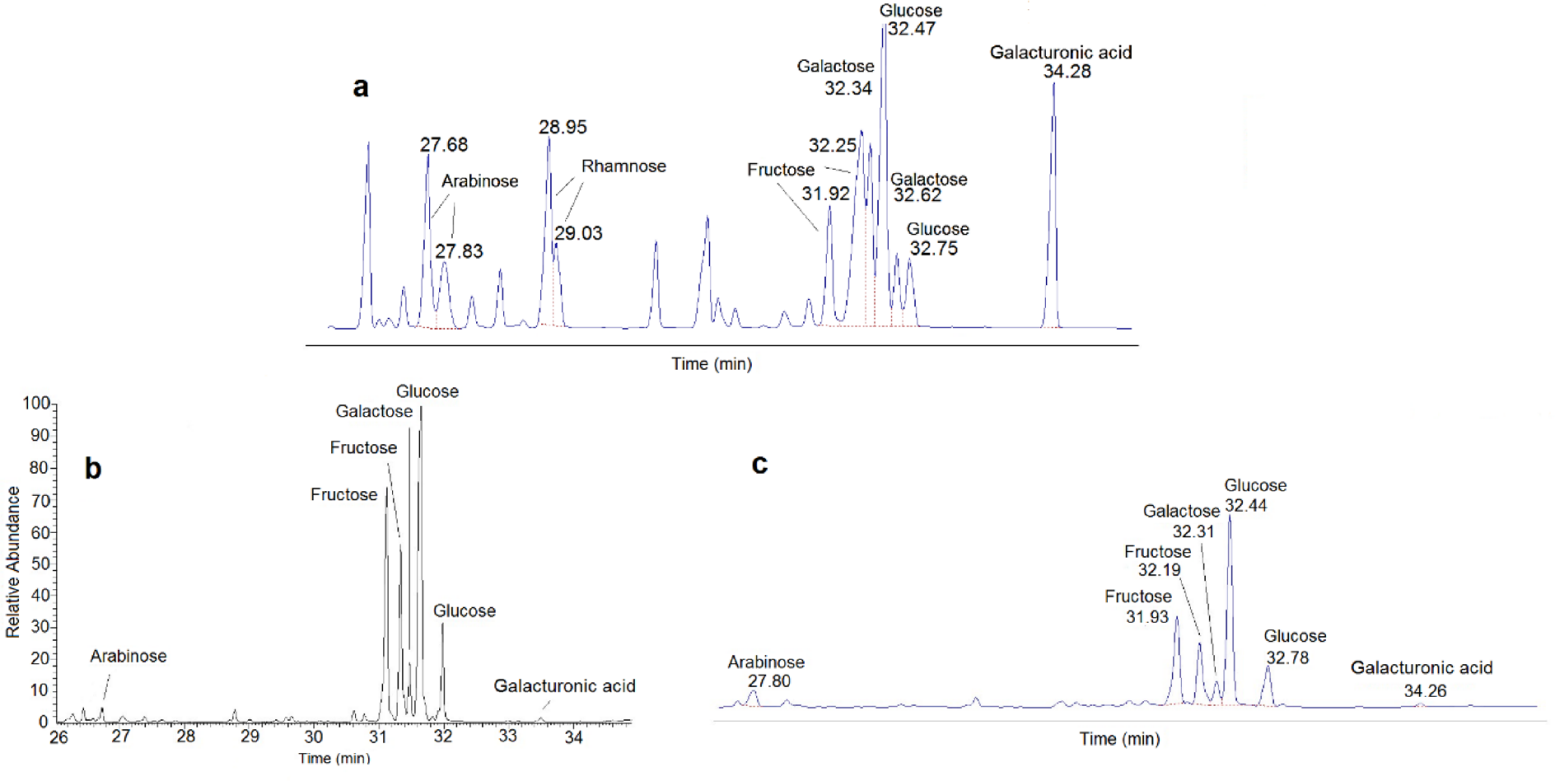
Gas chromatography results: (**a**) Standard monosaccharide profile by GC-FID; (**b**) EHC-1 analysis by GC–MS for identification of monosaccharides; (**c**) EHC-1 analysis by GC-FID for quantification of monosaccharides.

The relative monosaccharide composition of EHC-1 was determined through acid hydrolysis followed by derivatization analysis using GC–MS (for qualitative identification) and GC-FID (for quantification). To ensure high quantitative accuracy, the peak areas of both syn and anti isomers were quantified collectively for each monosaccharide. The final composition was calculated using Relative Response Factors (RRFs) to account for the varying FID responses of the different monosaccharide derivatives. The combined chromatographic analysis (Fig. 4a–c) revealed that EHC-1 is composed of glucose (51.50%), fructose (35.70%), galactose (5.79%), arabinose (5.86%), and galacturonic acid (1.13%).

### Analysis of EHC-1: X-ray diffraction

**Fig. 5:**
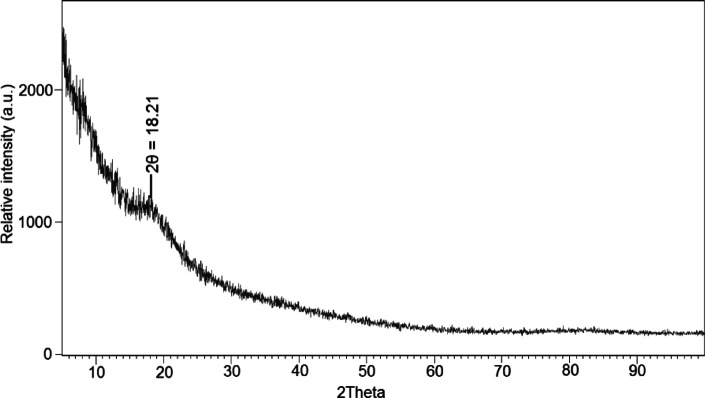
XRD pattern of EHC-1.

The XRD profile of EHC-1 (Fig. 5) exhibited a diffraction peak at 2θ = 18.21**°**, along with an extensive amorphous region. The crystallinity index was determined to be 42%, confirming that the EHC-1 possesses a semi-crystalline structure with a predominantly amorphous nature.

### Analysis of EHC-1: thermal behavior

**Fig. 6:**
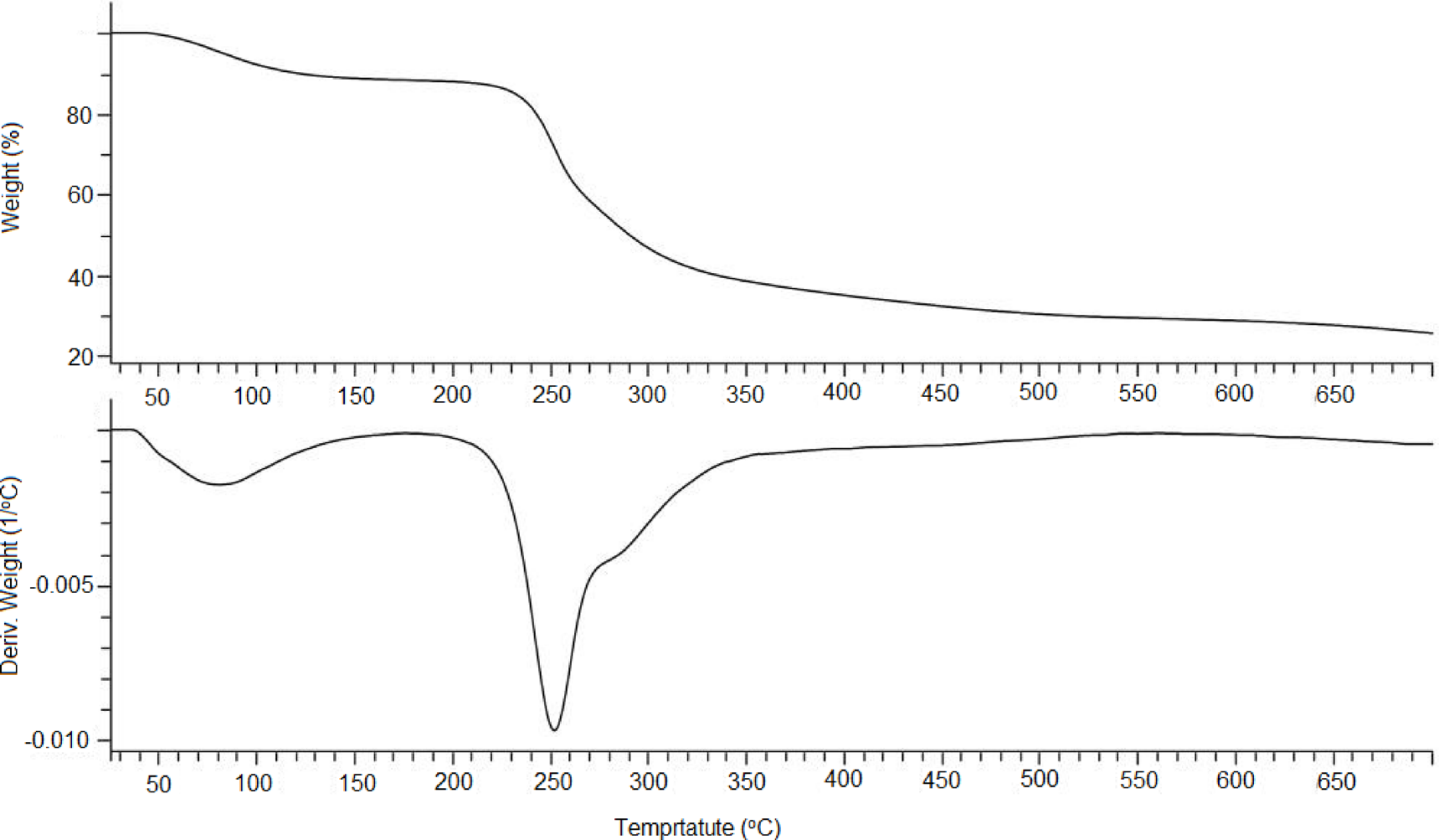
Thermal behavior (TGA and DTG) of EHC-1.

Thermogravimetric analysis (TGA) of EHC-1 was conducted on a Mettler Toledo instrument from 25 to 700 °C at 10 °C/min under an argon atmosphere. The TGA curve (Fig. 6) revealed two main stages of mass loss. The first phase, occurring below 100 °C, was associated with a minor weight loss due to the desorption of physically bound water^27,28^. The second major degradation phase occurred between 200 and 350 °C, with a maximum decomposition rate observed at approximately 250 °C, leading to 62.8% weight loss. This substantial mass loss indicates thermal decomposition of the polysaccharide backbone, resulting from depolymerization and the cleavage of glycosidic bonds. A gradual weight loss was observed between 400 to 700 °C, reflecting the degradation of residual organic components.

### Antioxidant activity of EHC-1

#### DPPH radical scavenging assay

**Fig. 7:**
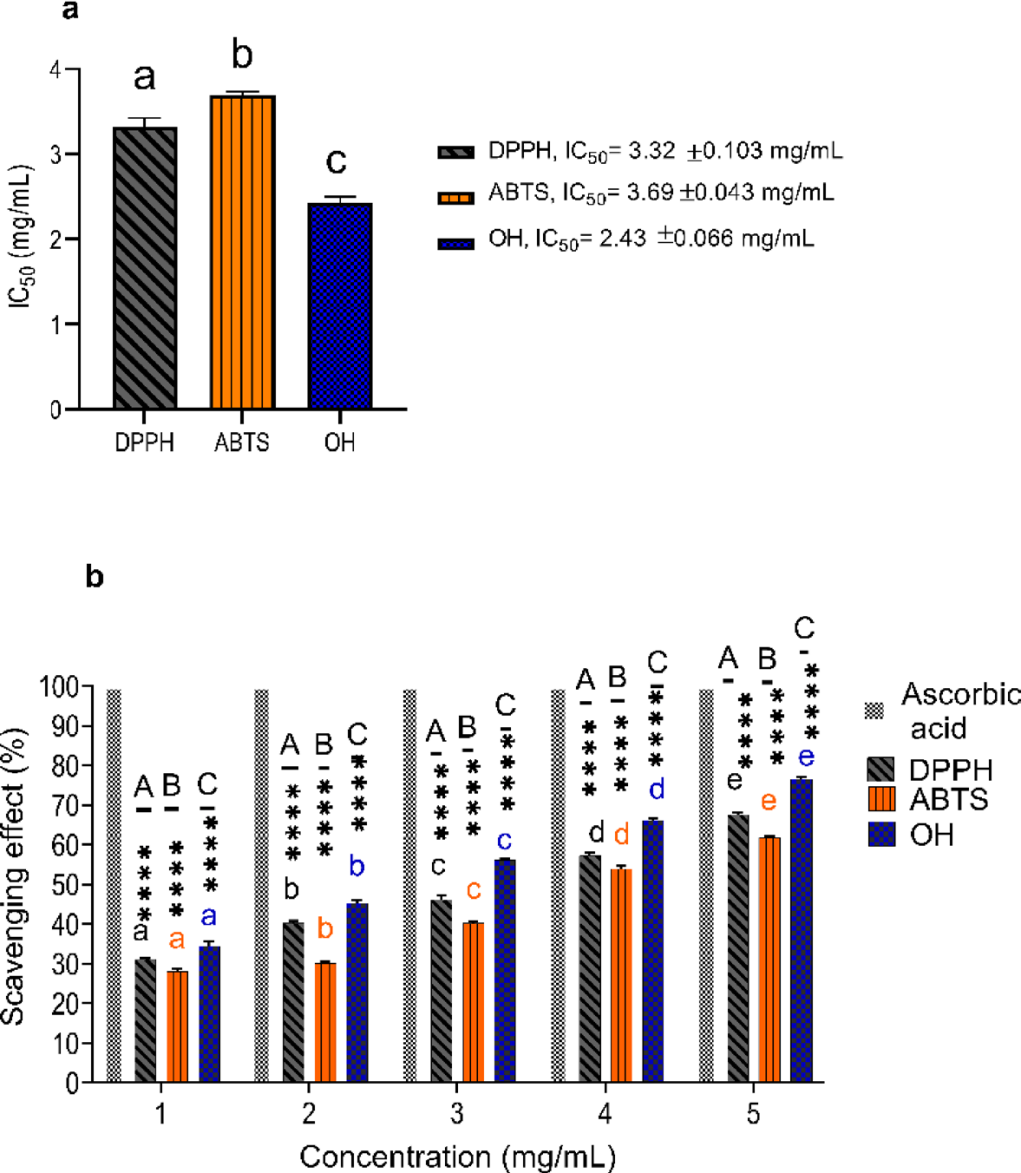
Antioxidant activities of EHC-1. (**a**) Comparison of IC_50_ values (mg/mL) for DPPH, ABTS, and hydroxyl (OH) radical scavenging activities; different lowercase letters (a–c) indicate significant differences between the assays (*p* < 0.0001) based on one-way ANOVA; (**b**) Dose-dependent radical scavenging effects of EHC-1 compared to ascorbic acid (positive control). Results are expressed as mean ± SD (n = 4). In panel (**b**) different lowercase letters (a–e) of the same color indicate significant differences between concentrations within each radical type (*p* < 0.0001); different uppercase letters (A–C) at the same concentration indicate significant differences between the radical types (*p* < 0.0001); asterisks (****) denote significant differences between EHC-1 concentrations and the positive control (*p* < 0.0001) as determined by two-way ANOVA followed by Tukey’s post-hoc test.

The DPPH assay was employed to evaluate the radical scavenging capacity of EHC-1 by measuring the absorbance decrease at 517 nm (Fig. 7a, b)^29^. At the highest tested concentration (5.0 mg/mL), EHC-1 exhibited a scavenging activity of 67.58 ± 0.51%, whereas ascorbic acid (as the positive control) showed 100% activity. Statistical analysis revealed that EHC-1 exhibited lower scavenging activity compared to ascorbic acid at all tested concentrations (*p* < 0.0001, Fig. 7b). Furthermore, the scavenging activity of EHC-1 exhibited a clear dose-dependent trend, with statistically significant differences observed across all concentrations (*p* < 0.0001, indicated by lowercase black letters in Fig. 7b). At each concentration, the scavenging efficacy of EHC-1 against DPPH was different from the other two radicals (*p* < 0.0001, as indicated by the different uppercase letters A–C in Fig. 7b). The IC_50_ value for EHC-1 was calculated at 3.32 ± 0.10 mg/mL (Fig. 7a), indicating a moderate antioxidant activity.

#### ABTS radical scavenging assay

The ABTS radical scavenging assay was performed to determine the total antioxidant capacity of EHC-1 (Fig. 7a, b)^30^. The results demonstrated that the scavenging activity of EHC-1 increased in a dose-dependent manner across the tested concentrations. At the maximum concentration of 5.0 mg/mL, EHC-1 reached a scavenging efficacy of 61.83 ± 0.43%, which was lower than that of ascorbic acid (100%). Significant differences were observed among all evaluated concentrations of EHC-1 (*p* < 0.0001 indicated by lowercase orange letters in Fig. 7b), as well as between each concentration and the positive control (*p* < 0.0001, Fig. 7b). Moreover, ABTS scavenging activity was found to be the lowest among the three assays at most tested concentrations (*p* < 0.0001, indicated by uppercase letters in Fig. 7b). The IC_50_ value was calculated to be 3.69 ± 0.04 mg/mL (Fig. 7a).

#### Hydroxyl radical scavenging assay

The results of the OH radical scavenging activity of EHC-1 are illustrated in Figs. 7a, b. At a concentration of 5.0 mg/mL, EHC-1 exhibited an inhibition rate of 76.55 ± 0.55%, compared to 100% for ascorbic acid. Data analysis indicated that EHC-1 showed lower scavenging activity than the positive control across all tested concentrations (*p* < 0.0001, Fig. 7b). Additionally, a dose-dependent relationship was observed, with statistically significant differences between successive concentrations (*p* < 0.0001, indicated by lowercase blue letters in Fig. 7b). Notably, EHC-1 exhibited higher scavenging activity against OH radicals compared to DPPH and ABTS at all concentration levels (*p* < 0.0001, indicated by different uppercase letters A–C in Fig. 7b). The IC_50_ value was calculated as 2.43 ± 0.06 mg/mL. A comparative analysis of IC_50_ values further confirmed that EHC-1 possessed the highest scavenging activity against hydroxyl radicals (*p* < 0.0001, Fig. 7a).

### Antibacterial activity of EHC-1

The antibacterial activity of EHC-1 was investigated against *E. coli* ATCC 11,775 (Gram-negative) and *S. aureus* ATCC 12,600 (Gram-positive) using the broth microdilution method. The minimum inhibitory concentration (MIC) and minimum bactericidal concentration (MBC) values are reported in Table 1. EHC-1 exhibited MIC values of 12.5 mg/mL against *E. coli* and 6.25 mg/mL against *S. aureus*, while the MBC was recorded at 12.5 mg/mL for both strains.

**Table 1:** In vitro antibacterial activity of the EHC-1.

Sample	*Escherichia coli* ATCC 11,775		*Staphylococcus aureus* ATCC 12,600	
	MIC (mg/mL)	MBC (mg/mL)	MIC (mg/mL)	MBC (mg/mL)
EHC-1	12.5	12.5	6.25	12.5
Cefixime	0.0005	0.001	0.004	0.016

## Discussion

In the present study, a purified polysaccharide (EHC-1) was successfully isolated and partially characterized from *E. hebecarpa*. Monosaccharide relative composition analysis revealed that EHC-1 is rich in glucose, which aligns with previously reported polysaccharides isolated from *E. fischeriana* and *E. himalayensis*^17,18^. However, notable differences were observed in the detailed monosaccharide profiles and relative molar ratios among these three species. For example, similar to *E. himalayensis*, EHC-1 contained glucose and galactose but lacked detectable mannose^18^. Furthermore, while EHC-1 exhibited a monosaccharide diversity comparable to that of *E. fischeriana*, mannose was also absent in EHC-1^17^. Consistent with reports on *E. humifusa*, EHC-1 included arabinose, galactose and galacturonic acid, whereas rhamnose was not detected^31^. These compositional disparities are primarily attributable to interspecific variability, reflecting species-specific metabolic pathways and genetic frameworks.

From a structural perspective, EHC-1 showed a semi-crystalline nature, a characteristic consistent with carbohydrates extracted from diverse sources such as *Althaea officinalis* L. root, almond gum, and dandelion root^32–34^. Notably, EHC-1 exhibited a higher crystallinity index compared to that of carbohydrates derived from almond gum. Such variations in crystallinity are likely ascribed to differences in the botanical origin and solubility properties of the respective polysaccharides^27,32^. The thermal stability of EHC-1, characterized by a maximum degradation temperature (T_max_) at approximately 250 °C, was consistent with those reported for polysaccharides from *Ophiopogon japonicus* root (283 °C), *Althaea officinalis* L. root (~ 300 °C), and coffee polysaccharides (~ 300 °C)^28,33,35^. These similarities suggest that the thermal behavior of EHC-1 is influenced by its monosaccharide composition, molecular aggregation, and intermolecular interactions.

The antioxidant efficacy of polysaccharides is influenced by several factors, including molecular weight, uronic acid content, monosaccharide composition, and conformational features^36^. These compounds may exert antioxidant effects through several mechanisms. First, polysaccharides may function as radical scavengers by donating electrons or hydrogen atoms to neutralize free radicals^37^. Second, they can chelate transition metal ions, such as iron, thereby inhibiting metal-catalyzed radical formation. Third, polysaccharides can enhance endogenous antioxidant defense systems by upregulating the activities of enzymes such as superoxide dismutase (SOD), catalase (CAT), and glutathione peroxidase (GSH-Px)^38^. In the present study, EHC-1 exhibited moderate antioxidant activity. This behavior could be attributed to its relatively high molecular weight (148 kDa). Generally, lower-molecular-weight polysaccharides demonstrate superior antioxidant activity due to the greater availability of reducing terminal hydroxyl groups for quenching free radicals^39^. Furthermore, the uronic acid content of EHC-1 was low. Uronic acids are known to enhance antioxidant capacity by facilitating hydrogen release from O–H bonds due to their electrophilic nature^39^. Additionally, the absence of rhamnose and mannose, coupled with the predominance of glucose and fructose, may underlie the observed moderate antioxidant activity^36,39^. The high purity of EHC-1 also suggests minimal interference from non-carbohydrate impurities, such as proteins and phenolic compounds, which are often associated with enhanced antioxidant effects^39^. Notably, the high crystallinity of EHC-1 might reflect a robust intramolecular hydrogen bonding network, which limits the accessibility of hydroxyl groups essential for effective radical scavenging. Compared with polysaccharides derived from the roots of *E. himalayensis* (IC_50_ = 0.224 µg/mL for DPPH), EHC-1 displayed lower antioxidant potency (IC_50_ = 3.32 mg/mL). This disparity is likely attributable to its lower uronic acid content and higher molecular weight^18^. Similarly, the scavenging activity of EHC-1 was less pronounced than that of *E. bivonae* polysaccharides (IC_50_ < 1 mg/mL), presumably due to variations in molecular conformation and monosaccharide profiles^20^. In comparison with well-characterized polysaccharides, such as sulfated fucoidan (IC_50_ = 0.43 mg/mL for DPPH)^40^, EHC-1 demonstrated lower efficacy. This may be ascribed to the absence of sulfate and uronic acid groups. Furthermore, EHC-1 exhibited reduced activity relative to *Ganoderma lucidum* polysaccharides (IC_50_ < 0.5 and 1.5 mg/mL for DPPH and ABTS, respectively)^41^, possibly owing to the lower molecular weight and distinct structural features inherent in *Ganoderma* species. The moderate ABTS radical scavenging activity of EHC-1 was consistent with its DPPH scavenging behavior. Overall, these findings suggest that EHC-1 possesses a stronger hydroxyl radical scavenging potential compared to its efficacy against DPPH and ABTS radicals.

This study provides the first report on the antibacterial potential of a purified polysaccharide (EHC-1) isolated from *E. hebecarpa*. The antimicrobial activity of polysaccharides is influenced by several factors, including monosaccharide composition, molecular weight, functional groups, and molecular conformation. Generally, polysaccharides with lower molecular weight exhibit higher solubility and a superior ability to penetrate bacterial cell walls, enabling interactions with membrane proteins and subsequent disruption of membrane permeability. Moreover, uronic acids enhance interactions with bacterial surfaces, potentially interfering with adhesion and colonization. Carboxyl groups further increase the capacity of polysaccharides to interact with the positively charged bacterial membrane through electrostatic attraction, leading to membrane disruption^42^. In the case of EHC-1, its moderate-to-weak antibacterial activity can be explained by its relatively high molecular weight (148 kDa) and low uronic acid content, despite its high glucose and fructose proportions. The higher sensitivity of *S. aureus* (Gram-positive) compared to *E. coli* (Gram-negative) suggests that the absence of an outer membrane in Gram-positive bacteria may facilitate the penetration of EHC-1, thereby enhancing its bactericidal action. Compared with other plant-derived polysaccharides, EHC-1 exhibited a lower antibacterial profile. Its potency was lower than that of fucoidan isolated from *Sargassum polycystum*, with reported MIC and MBC values of 200 and 300 µg/mL against *E. coli* and *S. aureus*, respectively^43^. Similarly, EHC-1 (MIC: 12.5 and 6.25 mg/mL) showed significantly weaker inhibitory effects than alginate oligosaccharides (MIC: 0.312 and 0.016 µg/mL against *E. coli* and *S. aureus*)^44^. Furthermore, the antibacterial activity of EHC-1 was considerably less pronounced than that of chitosan, which typically yields MIC values below 2 mg/mL under comparable experimental conditions and a pH range of 4.5 to 7.5^45^. The relationship between MIC and MBC provides valuable insights into the nature of the antimicrobial effect. In this study, the MBC/MIC ratios for *E. coli* and *S. aureus* were determined to be 1 and 2, respectively. According to previous studies, an antimicrobial agent is typically classified as bactericidal when the MBC/MIC ratio is ≤ 4^46,47^. Therefore, these results suggest that EHC-1 may exert a bactericidal rather than a bacteriostatic effect against the tested strains.

## Conclusion

This research successfully isolated and purified EHC-1, a water-soluble polysaccharide, from *E. hebecarpa* roots. A partial characterization using GC-FID, GC-MS, XRD, TGA, and GPC revealed that EHC-1 is primarily a heteropolysaccharide composed of glucose, fructose, galactose, arabinose, and galacturonic acid. Structurally, EHC-1 showed a semi-crystalline architecture with a significant amorphous proportion, while its thermal behavior demonstrated structural degradation at approximately 250 °C. In vitro assays revealed that EHC-1 possesses moderate antioxidant activity, with its scavenging efficacy against hydroxyl radicals being slightly higher than that observed for DPPH and ABTS. Moreover, EHC-1 exhibited moderate antibacterial properties against *E. coli* and *S. aureus*. To fully harness the functional potential of EHC-1, future studies should focus on in-depth structural elucidation. Specifically, the employment of advanced NMR techniques (^1^H, ^13^C, and 2D-NMR) will be indispensable in future investigations to achieve a definitive and full structural characterization. Furthermore, rheological investigations to assess viscosity and gelation properties for industrial applications, along with exploring the synergistic effects of EHC-1 with other natural antimicrobial agents, are warranted. Finally, enhancing its biological activity through chemical modifications (e.g., sulfation or selenization) remains a promising avenue to broaden its therapeutic and functional applications.

## Materials and methods

### Plant material

Roots of *E. hebecarpa* were collected in June 2019 from Sepidan County, Fars Province, Iran (30° 17 ′N, 51° 55 ′E, altitude 2478 m). Collection was conducted in accordance with World Health Organization (WHO) guidelines on good agricultural and collection practices (GACP) for medicinal plants. The plant was taxonomically authenticated by Dr. Mojtaba Asadollahi, and a voucher specimen (No. MPH-3232) was deposited in the Herbarium of the Medicinal Plants and Drugs Research Institute, Shahid Beheshti University, Tehran, Iran.

*E. hebecarpa* is a common and widely distributed plant and is not an endangered species. The collection for academic study was performed under the official permission of the university and complied with international guidelines for collecting plants, including the IUCN Policy Statement on Research Involving Species at Risk of Extinction and the Convention on the Trade in Endangered Species of Wild Fauna and Flora.

### Materials and chemicals

Monosaccharide standards, DPPH, ABTS and methoxyamine hydrochloride were purchased from Sigma Chemical Co. (St. Louis, MO, USA). Trimethylchlorosilane, bis- (O, N)-trimethylsilyltrifluoroacetamide (BSTFA), iron(II) sulfate, Trifluoroacetic acid (TFA), pyridine, hydrogen peroxide, potassium persulfate, and salicylic acid were obtained from Merck Co (Darmstadt, Germany). DEAE-Cellulose A52 and Sephadex G-200 were supplied from the Pharmacia Co. (Uppsala, Sweden). Hydrochloric acid (37%), n-butanol, and chloroform were purchased from Carlo Erba Co (Val-de-Reuil, France).

### Isolation of the *E. hebecarpa* polysaccharide

To isolate polysaccharides from *E. hebecarpa*, 150 g of roots were thoroughly cleaned to remove impurities and subjected to three successive reflux extractions with 1500 mL of 96% (v/v) ethanol for 1 h each. After each extraction cycle, the ethanolic supernatant was discarded to remove non-polar impurities. The residual plant material was subsequently extracted three times with 1500 mL of distilled water at 75 °C for 1 h per extraction. All resulting aqueous extracts were combined, filtered, and concentrated using a Heidolph Laborota 4000 rotary evaporator. The concentrated aqueous extract was combined with four volumes of ethanol and stored at 4 °C for 24 h to facilitate polysaccharide precipitation. The resulting precipitate was collected via centrifugation (4000 rpm for 20 min). Protein impurities were subsequently removed through three successive treatments using the Sevag method (n-butanol/chloroform, 1:4 v/v)^21^. The resulting protein-free solution was freeze-dried to yield 2.1 g of CEHC. The CEHC was dissolved in deionized water, filtered through a 0.45 μm membrane, and loaded onto a DEAE-52 cellulose column (2.6 × 30 cm). Elution was performed using a stepwise gradient of NaCl solutions (0.1, 0.2, 0.3, and 0.4 M) at a constant flow rate of 2.5 mL/min^48^. Carbohydrate-containing fractions (each 4 mL) were monitored at 490 nm using the phenol–sulfuric acid method^49^. Fractions 33–43, which exhibited the highest carbohydrate content, were pooled (375 mg, designated EHC). This fraction was further purified on a Sephadex G-200 column (1.6 cm × 40 cm) with 0.1 M NaCl as the eluent^50^.

Fractions 38–53, corresponding to the main elution peak, were combined and subjected to ultrafiltration using an Amicon® centrifugal filter with a 3 kDa molecular weight cutoff (MWCO). The fraction with molecular weights above 3 kDa was then collected and lyophilized, yielding 160 mg of the purified polysaccharide (EHC-1) for further structural and compositional analyses.

### Molecular weight analysis of EHC-1

The molecular weight distribution of EHC-1 was analyzed by high-performance liquid chromatography (HPLC) using a Waters Ultrahydrogel 250 Å column (7.8 mm × 300 mm, USA) connected to a Shimadzu LC-20A system equipped with a refractive index detector (RID). Elution was performed with 0.1 M NaNO_3_ at a flow rate of 1 mL/min, maintaining the column temperature at 35 °C^48^.

### Spectroscopic analysis

UV–Vis analysis was conducted using a Shimadzu UV-2501PC spectrophotometer (Japan) to detect the presence of proteins and nucleic acids based on their characteristic absorption at 280 nm and 260 nm, respectively^51^. FT-IR spectra were recorded on a Tensor 27 spectrometer (Bruker, USA). Samples were prepared as KBr pellets, and spectra were collected over the range of 4000 to 400 cm^−1^^24^.

### Carbohydrate content and monosaccharide profile

The non-reducing carbohydrate content of EHC-1 was calculated by subtracting the reducing carbohydrate value from the total carbohydrate content. Total carbohydrate quantification was performed using the phenol–sulfuric acid method, with D-glucose employed as the calibration standard^49,52^. Reducing carbohydrate was assessed using the dinitrosalicylic acid method; and absorbance was recorded at 575 nm^53^. The purity of EHC-1 was estimated based on the ratio of its carbohydrate content to the total mass of EHC-1. Uronic acid content was determined using the m-hydroxybiphenyl colorimetric method, with the absorbance measured at 525 nm and D-galacturonic acid used as the standard^54,55^.

Monosaccharide composition analysis was performed following acid hydrolysis of 5 mg of EHC-1 using 2 M TFA at 120 °C for 90 min. The resulting monosaccharides were subsequently derivatized in a two-step procedure. Initially oxime derivatives were formed by reacting the sample with 50 μL of methoxyamine hydrochloride (20 mg/mL in pyridine) at 80 °C for 20 min. This was followed by silylation using 49.5 μL of N, O-bis(trimethylsilyl)trifluoroacetamide (BSTFA) and 1 μL of trimethylchlorosilane (TMCS), with heating at 80 °C for an additional 20 min.

GC-FID analysis was performed using a Thermoquest-Finnigan instrument equipped with a DB-5 fused-silica capillary column (60 m × 0.25 mm ID × 0.25 μm). The oven temperature was programmed to start at 60 °C (held for 5 min), increase at 5 °C/min to 280 °C, and maintain at this temperature for 11 min. Nitrogen was used as the carrier gas at a flow rate of 1.1 mL/min, while detector and injector temperatures were set at 280 °C and 250 °C, respectively. For monosaccharide identification, GC–MS was conducted on a Thermoquest-Finnigan Trace system under identical chromatographic conditions, with Helium as the carrier gas. Monosaccharides were identified by comparing their mass spectra and retention index (RI) with data from the Golm Metabolome Database (GMD) and authentic trimethylsilyl (TMSi) standards^24^. Since the formation of oxime derivatives involves the creation of a C=N bond, this derivatization typically yields syn and anti isomers for each monosaccharide^56^. For quantification, the relative composition was determined using the corrected peak area method. Relative Response Factors (RRFs) were first calculated from an external standard mixture containing each monosaccharide at known concentrations, with glucose serving as the reference (RRF = 1.0)^57^. For the hydrolyzed polysaccharide sample, the total peak area of the isomers for each monosaccharide was multiplied by its corresponding RRF. The final relative percentages were then calculated by dividing these corrected areas by the sum corrected area of all identified monosaccharides.

### X-ray diffractometry analysis

The crystalline structure of EHC-1 was investigated using XRD. Measurements were performed on an X’Pert PRO MPD diffractometer (PANalytical BV). The instrument utilized a Cu kα radiation (λ = 1.54 Å) source, operating at 40 mA and 40 kV. Diffraction patterns were recorded over a 2θ angle range of 5–100°, with a step size of 0.0260°. The crystallinity index was subsequently calculated following the formula^28^:

1 $${\text{Crystallinity Index}}\left( \% \right) = \left[ {\frac{{{\mathrm{Crystallinearea}}}}{{\left( {{\text{crystallinearea }} + {\text{ amorphousarea}}} \right)}} \, } \right] \times {1}00$$

### Thermal behavior

The thermal stability and degradation behavior of EHC-1were investigated using TGA on a Mettler Toledo thermal analysis instrument (Switzerland). Approximately 5 mg of sample was accurately weighed into an alumina crucible and heated from 25 to 700 °C at a constant rate of 10 °C/min under a continuous argon flow to prevent oxidation.

### Evaluation of antioxidant activities

#### DPPH radical scavenging capability

The DPPH radical scavenging activity of EHC-1 was evaluated using a modified method described by Li et al.^58^. Briefly, EHC-1 solutions were prepared in distilled water at various concentrations of 0 (negative control) and 1, 2, 3, 4, and 5 mg/mL. For each reaction, 50 μL of the EHC-1 solution was mixed with 200 μL of a 0.0788 mg/mL DPPH methanolic solution. The mixture was incubated in the dark at room temperature for 30 min. Absorbance was measured at 517 nm using a spectrophotometer. Ascorbic acid was used as a positive control. The DPPH radical scavenging activity was calculated using following formula:

2 $${\text{Scavenging activity}}\left( \% \right) = \left[ {\frac{{\left( {{\mathrm{Abs}}_{{{\mathrm{control}}}} {-}{\text{ Abs}}_{{{\mathrm{sample}}}} } \right)}}{{{\mathrm{Abs}}_{{{\mathrm{control}}}} }}} \right] \times {1}00$$

#### ABTS radical scavenging activity

The ABTS radical cation scavenging capacity of EHC-1 was assessed following previously reported methods^59,60^. The ABTS radical cation working solution was prepared by mixing 7.0 mM ABTS with 2.45 mM potassium persulfate and allowing the mixture to react in the dark at room temperature for 16 h. Prior to analysis, the ABTS radical solution was diluted with 10 mM PBS (pH 7.4) to achieve an absorbance of 0.700 ± 0.020 at 734 nm. In each reaction, 10 μL of the sample was added to 290 μL of diluted ABTS solution. After incubation for 10 min, absorbance was measured at 734 nm. Ascorbic acid was used as a reference antioxidant. The ABTS radical scavenging efficacy was calculated using the same equation applied for the DPPH assay.

#### Hydroxyl radical scavenging capability

Hydroxyl radical (·OH) scavenging activity of EHC-1 was assessed based on the method described by Guan et al., with minor modifications^61^. Briefly, 1 mL aliquots of the EHC-1 solutions (1–5 mg/mL) were mixed with 1 mL of 6 mM FeSO4 and 1 mL of 6 mM salicylic acid in ethanol. After standing at room temperature for 10 min, 1 mL of 2.4 mM hydrogen peroxide was added, and the mixture was incubated at 37 °C for 30 min in the dark. Absorbance was subsequently measured at 510 nm. Ascorbic acid served as the positive control. Hydroxyl radical scavenging activity was calculated using the following equation:

3 $${\text{Hydroxyl RSA}}\left( \% \right) = \left[ {{1}{-}\frac{{\left( {{\mathrm{A}}_{{\mathrm{S}}} - {\text{ A}}_{{\mathrm{B}}} } \right)}}{{{\mathrm{A}}_{{\mathrm{C}}} }}} \right] \times {1}00$$

where A_S_ is the absorbance of the sample solution, A_B_ is the absorbance of the solution without ethanol-salicylic acid, and A_C_ is the absorbance of the control containing no sample.

### Antibacterial activity

The antibacterial activity of EHC-1 was determined by evaluating the MIC and MBC against *S. aureus* ATCC 12,600 and *E. coli* ATCC 11,775 using the broth microdilution method in 96-well microplates, following Clinical and Laboratory Standards Institute (CLSI) guidelines, with minor modifications. Bacterial suspensions were adjusted to turbidity equivalent to the 0.5 McFarland standard using sterile saline and further diluted 1:100 prior to inoculation. EHC-1 solutions ranging from 0.03 to 20 mg/mL in Mueller–Hinton Broth (MHB) were added to the wells, followed by inoculation with bacterial suspensions. Plates were incubated at 37 °C for 22 h. MIC was defined as the lowest concentration at which no visible bacterial growth was observed. For MBC determination, 100 µL aliquots from wells without visible growth were subcultured onto agar plates and incubated at 37 °C for 24 h. MBC was defined as the minimum concentration of EHC-1 resulting in 99.9% bacterial cell death. Cefixime was simultaneously included as a positive control antibiotic^62^.

### Statistical analysis

All experiments were conducted in quadruplicate, and the data are presented as mean ± standard deviation (SD). Statistical analysis was performed using GraphPad Prism software (version 8.4.3). Differences in IC_50_ values (Fig. 7a) were evaluated using one-way analysis of variance (ANOVA). For the comparative evaluation of radical scavenging effects across different concentrations (Fig. 7b), a two-way ANOVA (factors: radical type and concentration) was employed. Both analyses were followed by Tukey’s post-hoc test for multiple comparisons. Statistical significance was set at *p* < 0.05.

## Data Availability

The datasets used and/or analyzed during the current study are available from the corresponding author upon reasonable request.
